# Competition between drum and quasi-planar structures in RhB_18_^–^: motifs for metallo-boronanotubes and metallo-borophenes†
†Electronic supplementary information (ESI) available: The photoelectron spectrum of RhB_18_^–^ at 266 nm; the top 42 low-lying isomers of RhB_18_^–^; the relative Gibbs free energies, valence molecular orbitals, and coordinates of the two most stable isomers of RhB_18_^–^. See DOI: 10.1039/c6sc02623k



**DOI:** 10.1039/c6sc02623k

**Published:** 2016-07-25

**Authors:** Tian Jian, Wan-Lu Li, Xin Chen, Teng-Teng Chen, Gary V. Lopez, Jun Li, Lai-Sheng Wang

**Affiliations:** a Department of Chemistry , Brown University , Providence , Rhode Island 02912 , USA . Email: Lai-Sheng_Wang@brown.edu; b Department of Chemistry and Key Laboratory of Organic Optoelectronics & Molecular Engineering of Ministry of Education , Tsinghua University , Beijing 100084 , China . Email: junli@tsinghua.edu.cn

## Abstract

Metal-doped boron clusters provide new opportunities to design nanoclusters with interesting structures and bonding. A cobalt-doped boron cluster, CoB_18_^–^, has been observed recently to be planar and can be viewed as a motif for metallo-borophenes, whereas the *D*_9d_ drum isomer as a motif for metallo-boronanotubes is found to be much higher in energy. Hence, whether larger doped boron drums are possible is still an open question. Here we report that for RhB_18_^–^ the drum and quasi-planar structures become much closer in energy and co-exist experimentally, revealing a competition between the metallo-boronanotube and metallo-borophene structures. Photoelectron spectroscopy of RhB_18_^–^ shows a complicated spectral pattern, suggesting the presence of two isomers. Quantum chemistry studies indicate that the *D*_9d_ drum isomer and a quasi-planar isomer (*C*_s_) compete for the global minimum. The enhanced stability of the drum isomer in RhB_18_^–^ is due to the less contracted Rh 4d orbitals, which can have favorable interactions with the B_18_ drum motif. Chemical bonding analyses show that the quasi-planar isomer of RhB_18_^–^ is aromatic with 10 π electrons, whereas the observed RhB_18_^–^ drum cluster sets a new record for coordination number of eighteen among metal complexes. The current finding shows that the size of the boron drum can be tuned by appropriate metal dopants, suggesting that even larger boron drums with 5d, 6d transition metal, lanthanide or actinide metal atoms are possible.

## Abstract 2

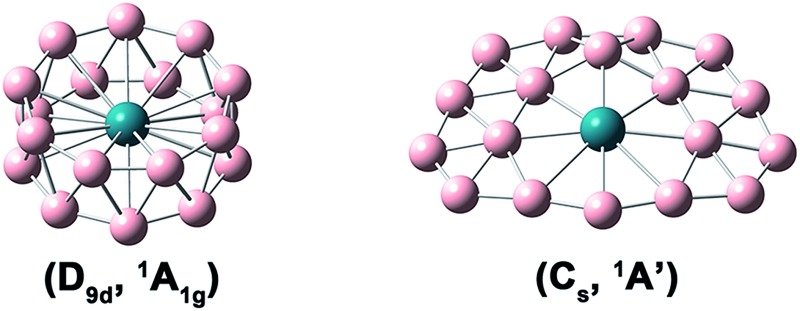
Two nearly degenerate isomers, one a drum and the other quasi-planar, are discovered for the gaseous RhB_18_^–^ cluster, revealing a competition between the metallo-boronanotube and metallo-borophene structures.

## 1. Introduction

The electron deficiency of boron has given rise to interesting structures and bonding in both elemental boron and boron compounds.^1,2^ Combined photoelectron spectroscopy (PES) and quantum chemistry theoretical studies over the past decade have also uncovered an interesting landscape for size-selected boron clusters (B_*n*_^–^) from planar structures to borospherene cages.^3–6^ The cationic boron clusters (B_*n*_^+^) have been found, by ion mobility experiment and theoretical calculations, to be planar up to *n* = 15 and tubular (or double-ring) for *n* > 15.^7^ Even though the B_20_ neutral boron cluster was first suggested to be tubular,^8^ an UV-IR double-resonance experiment failed to detect such structures.^9^ The discovery of the planar B_36_^–^ cluster with a hexagonal hole has provided indirect evidence for the viability of monolayer 2D borons, which we dubbed borophene.^10^ The recent experimental syntheses of borophenes on silver substrates,^11,12^ as proposed by theoretical calculations,^13–15^ have stimulated significant interests in the properties and structures of this new 2D boron material.

Heteroatom-doping can be used to modify and expand significantly the structures and properties of boron clusters. Inspired by the double aromaticity in the *D*_8h_ B_9_^–^ molecular wheel,^16^ a design principle has been proposed to produce transition metal centered borometallic molecular wheels.^17^ Combined PES and theoretical studies have characterized a series of these metal centered boron mono-wheels (M©B_*n*_^–^) with *n* ranging from 8 to 10.^18^ However, the metal-doped CoB_12_^–^ and RhB_12_^–^ clusters were found to have half-sandwich-like structures, with the metal atom bonded to the quasi-planar B_12_ motif.^19^ Computational studies have suggested that 3d-transition-metal-doped boron clusters MB_2*n*_ can form tubular (drum) structures with *n* from 6 to 8, but become cage-like structures with *n* from 9 to 10.^20,21^ Recently, joint PES and theoretical studies have shown that CoB_16_^–^ and MnB_16_^–^ indeed have drum structures with the metal atom sandwiched by two B_8_ rings and a record coordination number of sixteen.^22,23^ An interesting question is if larger metal-centered drum structures are still possible with even higher coordination numbers?

Very recently, a joint PES and theoretical investigation has shown that CoB_18_^–^ is a planar cluster with the Co atom being an integral part of the boron network, suggesting the possibility of metallo-borophenes,^24^ in which metal atoms are doped into the plane of borophenes.^25^ The putative CoB_18_^–^ drum isomer turns out to be much higher in energy, because the B_18_ tubular motif is too large to allow effective interactions between the Co 3d and the B 2p orbitals. Is it possible to design larger boron drums if transition metals with suitable sizes are used? A more important question is what governs the formation of drum structures or planar structures, which are motifs of metallo-boronanotubes and metallo-borophenes, respectively.

In the current work, we report a PES and theoretical investigation on RhB_18_^–^ to explore the possibility of a *D*_9d_ drum with an 18-coordinated Rh atom. PES of RhB_18_^–^ suggests the existence of isomers with a complicated spectral pattern. Global minimum searches along with calculations at different levels of density functional theory (DFT) and wavefunction theory (WFT) show that a perfect *D*_9d_ drum and a quasi-planar (*C*_s_) structure are nearly degenerate and are competing for the global minimum. The quasi-planar isomer is observed to be responsible for the main PES features, whereas the drum isomer corresponds to the weak PES features. Chemical bonding analyses show that the quasi-planar structure is aromatic with 10 delocalized π electrons. Significant covalent interactions are found between the Rh 4d and the B 2p orbitals, stabilizing the drum structure and pushing the limit of coordination number to eighteen. The current results show that there is a competition between quasi-planar structures and drum structures, depending on the size of the metal atoms and the bonding strength between them and the B atoms. Our results show that it is plausible to design metallo-boronanotubes and metallo-borophenes using different metal dopants with different sizes and bonding strengths.

## 2. Experimental method

The experiment was carried out using a magnetic-bottle PES apparatus equipped with a laser vaporization supersonic cluster source, details of which has been published elsewhere.^26^ The RhB_18_^–^ cluster was generated by laser vaporization of a Bi/^10^B/Rh composite target. The Bi component acted as a binder for the target preparation and also provided the Bi^–^ atomic anion for PES calibration. A helium carrier gas seeded with 5% argon was used to quench the plasma, initiating nucleation and cluster formation. Clusters formed in the nozzle were entrained in the carrier gas and went through a supersonic expansion to form a collimated cluster beam after a skimmer. Anion clusters were extracted from the molecular beam and analyzed by a time-of-flight mass spectrometer. The RhB_18_^–^ anion of interest was mass-selected, decelerated, and photodetached by the 193 nm (6.424 eV) radiation from an ArF excimer laser or the fourth harmonic radiation (266 nm, 4.661 eV) from a Nd:YAG laser. Photoelectrons were collected at nearly 100% efficiency by a magnetic bottle and analyzed in a 3.5 meter long electron flight tube. The spectra were calibrated using the known spectrum of Bi^–^ and the energy resolution of the apparatus was Δ*E*_k_/*E*_k_ ≈ 2.5%, that is, ∼25 meV for 1 eV electrons.

## 3. Theoretical methods

The global minimum structure of RhB_18_^–^ was searched using the TGMin code^10^ developed based on the constrained basin-hopping algorithm,^27^ which was done initially using the PBE exchange–correlation functional^28^ with the basis sets of double-ζ plus one polarization function (DZP)^29^ in the ADF 2013.01 program.^30^ Low-lying isomers were then re-optimized at both the PBE and hybrid PBE0 levels^31^ using the triple-ζ plus one polarization function (TZP) Slater-type basis sets^29^ to obtain refined relative energies. The small frozen core approximation was applied to the [1s^2^–3d^10^] core of Rh and the [1s^2^] core of B. Vibrational frequencies were computed for each isomer to verify the minimum on the potential energy surface. For the four lowest-lying isomers, we performed single-point WFT calculations at the level of coupled-cluster theory with single, double and perturbative triple excitations [CCSD(T)]^32,33^ using the MOLPRO 2012 software package.^34^ The geometries used in the CCSD(T) calculations were based on those obtained from the PBE0/TZP method. We used the all-electron triple-ζ basis set (cc-pVTZ) for B^35^ and the Stuttgart energy-consistent relativistic pseudo-potentials (ECP10MDF) with the corresponding ECP10MDF_VTZ basis set for Rh.^36^

The first ADEs and VDEs of the two lowest-lying isomers were calculated at the DFT level. Electron detachment energies from the ground state were calculated using the ΔSCF-TDDFT approach with the statistically averaged orbital potential (SAOP)^37^ *via* the ADF 2013.01 code, as outlined previously.^38^ The chemical bonding of the two most stable isomers was investigated using the adapted natural density partitioning (AdNDP) method^39^ at the PBE0 level of theory using cc-pVTZ and ECP10MDF_VTZ basis sets for B and Rh, respectively. The structural and chemical bonding pictures were visualized by GaussView.^40^

## 4. Experimental results

**Fig. 1:**
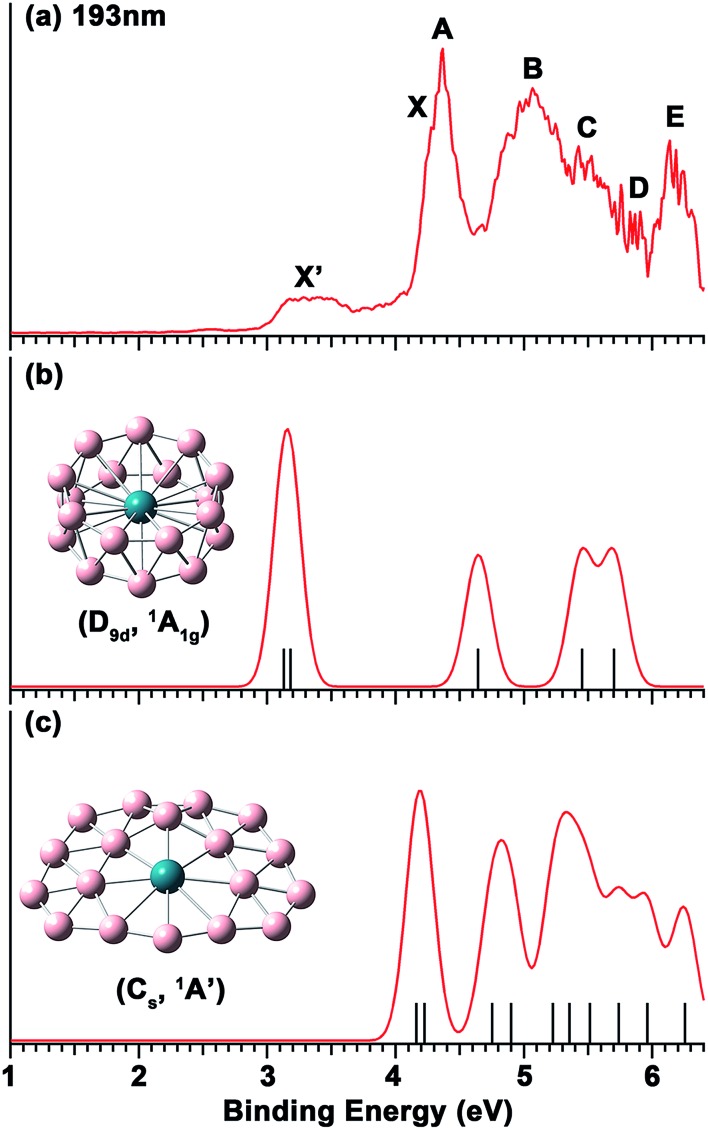
(a) The photoelectron spectrum of RhB_18_^–^ at 193 nm (6.424 eV). (b) Simulated spectrum for the *D*_9d_ isomer of RhB_18_^–^. (c) Simulated spectrum for the *C*_s_ quasi-planar isomer of RhB_18_^–^. The vertical bars in (b) and (c) represent the calculated VDEs given in Table 1. The simulated spectra were obtained by fitting the calculated VDEs with unit-area Gaussian functions of 0.1 eV half-width.

The photoelectron spectrum of RhB_18_^–^ at 193 nm shown in Fig. 1a displays a complicated spectral pattern with numerous broad detachment features. The relatively sharp and intense bands X and A are not resolved in the 193 nm spectrum, but can be seen more clearly in the 266 nm spectrum in Fig. S1.† Following an energy gap after band A, broad and continuous spectral features appear between 4.6 and 5.9 eV, suggesting a high density of final electronic states and possibly large geometry changes upon electron detachment. The B, C and D labels are given in this spectral range simply for the sake of discussion. At the high binding energy side, a well-separated and broad band E is observed at a vertical detachment energy (VDE) of 6.13 eV. The onset of band X yields an adiabatic detachment energy (ADE) of 4.10 eV. The measured VDEs for all the PES bands are summarized in Table 1, where they are compared with the theoretical data.

**Table 1:** Experimental VDEs (in eV) of RhB_18_^–^ compared with those calculated at the TD-DFT (SAOP)/TZP level of theory

Feature	VDE (exp.)	Electron configuration^*e*^	VDE (calc.)
***D***_**9d**_ **isomer**			
X′^*a*^	∼3.2	…5e_u_^4^5e_g_^4^6e_u_^4^6a_1g_^2^6e_g_^4^ **4a_2u_^1^**	3.13^*c*^
		…5e_u_^4^5e_g_^4^6e_u_^4^6a_1g_^2^ **6e_g_^3^**4a_2u_^2^	3.18
		…5e_u_^4^5e_g_^4^6e_u_^4^ **6a_1g_^1^**6e_g_^4^4a_2u_^2^	4.64
		…5e_u_^4^5e_g_^4^ **6e_u_^3^**6a_1g_^2^6e_g_^4^4a_2u_^2^	5.45
		…5e_u_^4^ **5e_g_^3^**6e_u_^4^6a_1g_^2^6e_g_^4^4a_2u_^2^	5.70

***C***_**s**_ **isomer**			
X^*b*^	4.25(5)	…16a′^2^11a′′^2^17a′^2^12a′′^2^18a′^2^13a′′^2^19a′^2^14a′′^2^15a′′^2^20a′^2^ **21a′** ^**1**^	4.16^*d*^
A	4.38(5)	…16a′^2^11a′′^2^17a′^2^12a′′^2^18a′^2^13a′′^2^19a′^2^14a′′^2^15a′′^2^ **20a′** ^**1**^21a′^2^	4.22
B	∼5.0	…16a′^2^11a′′^2^17a′^2^12a′′^2^18a′^2^13a′′^2^19a′^2^14a′′^2^ **15a′′** ^**1**^20a′^2^21a′^2^	4.75
		…16a′^2^11a′′^2^17a′^2^12a′′^2^18a′^2^13a′′^2^19a′^2^ **14a′′** ^**1**^15a′′^2^20a′^2^21a′^2^	4.90
C	∼5.5	…16a′^2^11a′′^2^17a′^2^12a′′^2^18a′^2^13a′′^2^ **19a′** ^**1**^14a′′^2^15a′′^2^20a′^2^21a′^2^	5.22
		…16a′^2^11a′′^2^17a′^2^12a′′^2^18a′^2^ **13a′′** ^**1**^19a′^2^14a′′^2^15a′′^2^20a′^2^21a′^2^	5.36
		…16a′^2^11a′′^2^17a′^2^12a′′^2^ **18a′** ^**1**^13a′′^2^19a′^2^14a′′^2^15a′′^2^20a′^2^21a′^2^	5.51
D	∼5.9	…16a′^2^11a′′^2^17a′^2^ **12a′′** ^**1**^18a′^2^13a′′^2^19a′^2^14a′′^2^15a′′^2^20a′^2^21a′^2^	5.74
		…16a′^2^11a′′^2^ **17a′** ^**1**^12a′′^2^18a′^2^13a′′^2^19a′^2^14a′′^2^15a′′^2^20a′^2^21a′^2^	5.96
E	6.13(6)	…16a′^2^ **11a′′** ^**1**^17a′^2^12a′′^2^18a′^2^13a′′^2^19a′^2^14a′′^2^15a′′^2^20a′^2^21a′^2^	6.25

^*a*^The first experimental ADE of band X′ is 2.98 ± 0.08 eV.

^*b*^The first experimental ADE of band X is 4.10 ± 0.06 eV.

^*c*^The first ADE was calculated to be 2.93 eV from PBE0/TZP calculations for the drum isomer.

^*d*^The first ADE was calculated to be 4.10 eV from PBE0/TZP calculations for the quasi-planar isomer.

^*e*^The orbitals for the electron-detachment are marked in bold face.

The weak and broad signals (X′) at the low binding energy side suggest that they come from a different isomer of RhB_18_^–^. At 266 nm, this part of the spectrum is not much better resolved, but almost continuous signals are observed (Fig. S1†). The higher binding energy detachment transitions of the isomer are likely buried in the signals of the main isomer. The first VDE and ADE are, respectively, estimated as ∼3.2 and 2.98 eV for the X′ band.

## 5. Theoretical results

To search for the global minimum structure of RhB_18_^–^, we generated more than 10 000 possible structures using the TGMin program at the PBE/DZP level of theory. Low-lying isomers were re-optimized at the PBE0/TZP and PBE/TZP levels of theory. Forty-two structures were found within 45 kcal mol^–1^ of the global minimum, as shown in Fig. S2.† Single-point energies at the CCSD(T)/B/cc-pVTZ/Rh/ECP10MDF_VTZ level of theory were also calculated for the four lowest-lying isomers to better establish the order of the relative energies.

At the PBE0/TZP level of theory, a perfect *D*_9d_ drum isomer I (*D*_9d_, ^1^A_1g_) was found to be most stable, with a quasi-planar isomer II (*C*_s_, ^1^A′) lying 4.90 kcal mol^–1^ higher. At the PBE/TZP level of theory, the *C*_s_ quasi-planar isomer II was found to be the global minimum, with the drum isomer I being 3.52 kcal mol^–1^ higher in energy. Using the optimized RhB_18_-clusters, the estimated binding energies of isomers I and II relative to Rh and unrelaxed B18- are 201.19 and 189.94 kcal mol^–1^, respectively, at the PBE level of theory. At the more accurate CCSD(T) level, isomer I was instead found to be the global minimum with isomer II lying 5.29 kcal mol^–1^ higher, which is similar to the PBE0 results. Thus, both isomers I and II were found to be rather close in energy, competing for the global minimum at different levels of theory. Since the two isomers have very different structures, entropy could play an important role in determining their energetic stability at finite temperatures. Thus, we also calculated the Gibbs free energies of these two isomers at the PBE0 level of theory from 100 to 1000 K, as shown in Fig. S3.† Apparently, the quasi-planar isomer II is favored entropically and becomes more stable than the drum isomer I above 650 K at the PBE0 level of theory. However, the relative energies of the two isomers are very close to each other in the whole temperature range and they could coexist in a wide range of temperatures. Therefore, these isomers are nearly degenerate and their relative energy is rather small, making it difficult to resolve the relative stability of the two isomers using approximate theoretical methods and truncated basis sets.

**Fig. 2:**
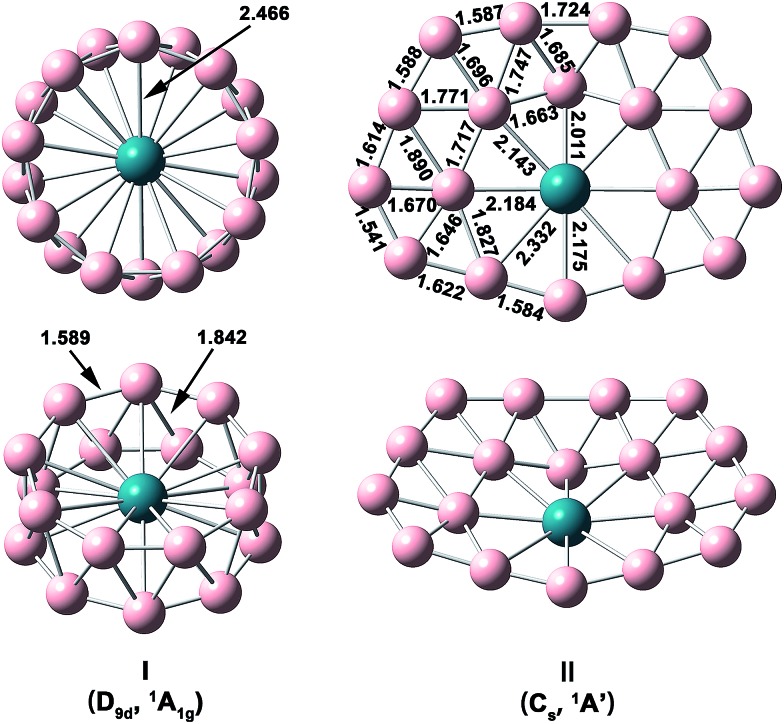
Structural details of the two lowest-lying isomers of RhB_18_^–^ at PBE0/TZP with their point-group symmetries and spectroscopic states. All distance are in Å.

The optimized structures and bond lengths of isomers I and II at the PBE0/TZP level are presented in Fig. 2. The B–B bond lengths in each B_9_ ring of the RhB_18_^–^ drum isomer is 1.59 Å, very close to the corresponding values in the Rh©B_9_ molecular wheel (1.54 Å)^41^ and the drum clusters CoB_16_^–^ (1.55–1.63 Å)^22^ and MnB_16_^–^ (1.58–1.62 Å).^23^ The structure of the quasi-planar isomer II is convex with the inner boron atoms buckled out. One of the inner B atoms is forced to be penta-coordinated and it exhibits the most significant buckling. The Cartesian coordinates of isomers I and II are given in Table S1.†

## 6. Comparison between experiment and theory

The calculated VDEs for the drum and quasi-planar isomers at the TD-DFT(SAOP)/TZP level are compared with the experimental data in Table 1, while the simulated spectra obtained by fitting a unit-area Gaussian function of 0.1 eV to each VDE are compared with the experimental spectrum in Fig. 1. Both isomers have closed-shell electron configurations and their valance molecular orbitals (MOs) are shown in Fig. S4 and S5.† With the single-particle approximation, one-electron detachment from each occupied valence orbital yields a single detachment channel, giving rise to relatively simple simulated spectra. The first and second computed VDEs of the quasi-planar RhB_18_^–^ are 4.16 and 4.22 eV, which are very close to each other and are in excellent agreement with the observed major detachment features X and A. There are eight more detachment channels below 6.3 eV for the quasi-planar isomer, consistent with the highly congested experimental spectrum.

The calculated first and second VDEs of the drum isomer of RhB_18_^–^ are also very close to each other and are in excellent agreement with the weak broad X′ feature at ∼3.2 eV. The HOMO–1 of the RhB_18_^–^ drum isomer (6e_g_, Fig. S4†) is degenerate; electron detachment from this MO would induce a strong Jahn–Teller effect, consistent with the broad width of the X′ band. There are three more detachment channels for the drum isomer within the experimental spectral range, but they are expected to be buried in the congested spectral features of the main quasi-planar isomer. Overall, the combined theoretical data for the two isomers are in good agreement with the experimental spectrum, lending considerable credence for the quasi-planar and drum structures identified theoretically as the two lowest lying isomers for RhB_18_^–^.

While the drum isomer is the global minimum at the CCSD(T) and PBE0 levels of theory it appears to be the minor isomer observed experimentally. This could be due to the entropical effect, *i.e.*, the quasi-planar isomer is more favored at finite temperatures. The experimental temperature of the RhB_18_^–^ cluster was not known, but should be below room temperature,^42^ at which the drum isomer is still more stable according to the PBE0 result (Fig. S3†). It is very likely that the relative energies of the two isomers are much closer than PBE0 or the approximate single-point CCSD(T) calculations suggested, *i.e.* the two isomers are essentially nearly degenerate on the basis of the current experimental results. As noted earlier, at the PBE level of theory, the quasi-planar isomer is indeed more stable than the drum isomer, consistent with the latter being the minor isomer.

## 7. Discussion

### 7.1. Chemical bonding in the drum structure of RhB_18_^–^

**Fig. 3:**
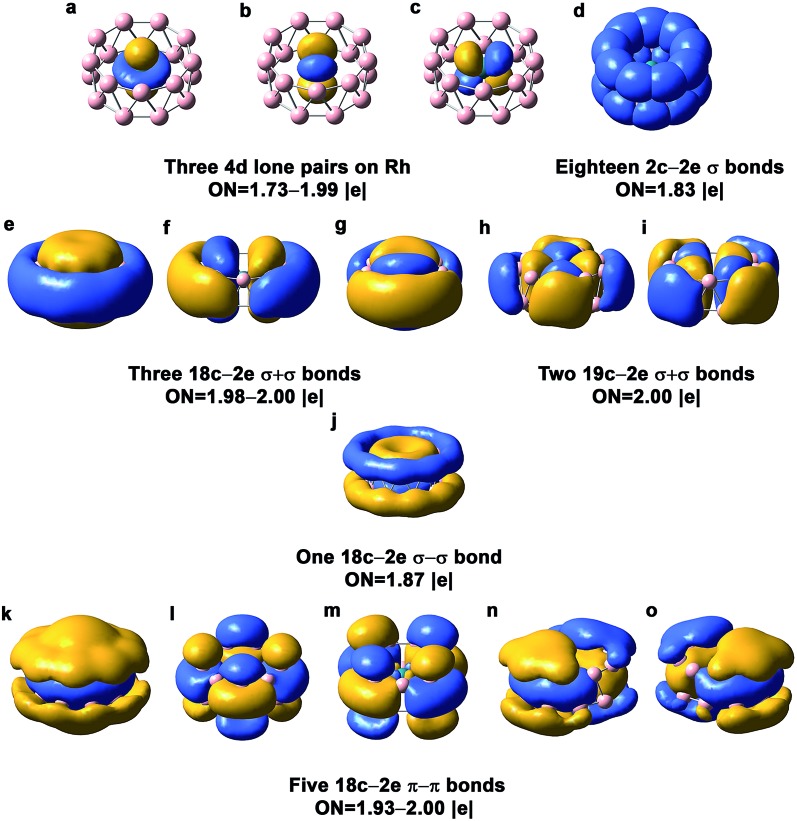
AdNDP chemical bonding analyses for the *D*_9d_ drum isomer of RhB_18_^–^ at the PBE0 level. ON stands for occupation number.

The chemical bonding in the drum and quasi-planar isomers of RhB_18_^–^ has been analyzed using the AdNDP method,^39^ which yields both localized and delocalized bonds simultaneously and provides chemically intuitive bonding pictures for complicated molecular systems. The chemical bonding in the RhB_18_^–^ drum isomer (Fig. 3) is reminiscent of the bonding pattern in the CoB_16_^–^ and MnB_16_^–^ drums.^22,23^ There are basically four different types of bonds in the drum isomer, corresponding to the four rows in Fig. 3. The first row displays essentially localized bonds, including the three out-of-plane Rh 4d electron pairs and eighteen localized 2c–2e B–B bonds on the two B_9_ rings. The occupation number (ON) of the Rh 4d_*z*^2^_ electron pair is 1.99|*e*|, suggesting little interaction with the B_18_ drum motif. The ON of the Rh 4d_*xz*_ and 4d_*yz*_ electron pairs is 1.73|*e*|, indicating that 0.27 electron from each Rh 4d orbital participates in weak π bonding with the B_18_ drum. The next three rows in Fig. 3 describe delocalized bonding between the two B_9_ rings or between the central Rh atom and the B_18_ drum motif. The “+” sign means that the delocalized bonds between the two B_9_ rings overlap positively, and *vice versa*. The second row shows three 18c–2e σ + σ bonds and two 19c–2e σ + σ bonds. The three 18c–2e bonds represent strong bonding interactions between the three delocalized σ bonds in each B_9_ ring, while the two 19c–2e bonds represent strong covalent bonding between the two in-plane Rh 4d orbitals (4d_*xy*_ and 4d_*x*^2^–*y*^2^_) and the B 2p orbitals on the B_18_ drum motif. These Rh and B_18_ bonding interactions can also be seen from the 4e_g_ MOs in Fig. S4,† where the 7e_g_ LUMO represents the antibonding interactions between the Rh 4d orbitals and the B_18_ drum motif. There is only one 18c–2e σ–σ bond shown in the third row of Fig. 3. The last row consists of five 18c–2e π–π bonds, representing bonding interactions between the delocalized π bonds on each B_9_ ring. There is significant charge transfer from Rh to the drum framework of B_18_, which was calculated to be 0.83 e based on the Mulliken population analysis at the PBE0 level.

It is interesting to compare the stabilities of the RhB_18_^–^ and CoB_18_^–^ drum isomers. While the B_18_ motif is similar in the two clusters, the less contracted 4d orbitals of Rh allow better overlap with the B 2p orbitals on the B_18_ motif than the Co 3d orbitals do, as revealed by the valence shell orbital radii of Co 3d (0.358 Å) and Rh 4d (0.604 Å).^43^ Although the bond length between Rh and B (2.47 Å) in the drum isomer is still longer than the single Rh–B bond (2.10 Å) according to the covalent radii for Rh and B proposed by Pyykkö,^44,45^ the high coordination number is sufficient to yield strong interactions between Rh and the B_18_ drum motif. On the other hand, the 2.47 Å Co–B bond length in the CoB_18_^–^ drum isomer is much longer than the single Co–B bond (1.96 Å), making it much less stable relative to the planar global minimum of CoB_18_^–^.

### 7.2. Chemical bonding in the quasi-planar structure of RhB_18_^–^

**Fig. 4:**
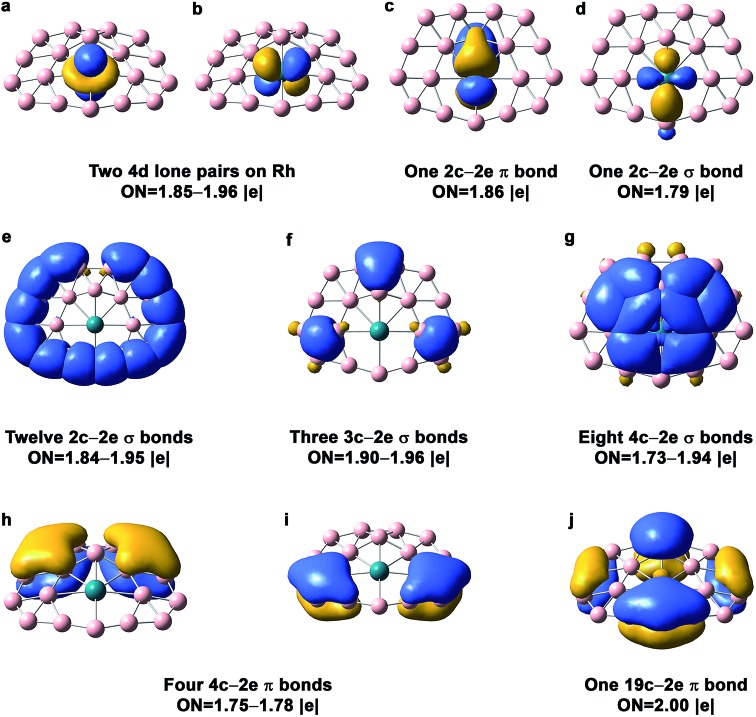
AdNDP chemical bonding analyses for the *C*_s_ quasi-planar isomer of RhB_18_^–^ at the PBE0 level. ON stands for occupation number.

The AdNDP bonding analysis for the quasi-planar RhB_18_^–^ is shown in Fig. 4. The first row in Fig. 4 includes two 4d lone pairs on Rh (4d_*z*^2^_, 4d_*xz*_) and localized π and σ bonding between Rh and two of its neighboring B atoms. It is interesting to see that there are only twelve 2c–2e peripheral B–B σ bonds (Fig. 4e). The long peripheral B–B bond (1.724 Å, Fig. 2) corresponds to a 3c–2e σ bond, as shown in Fig. 4f, which also contains two additional 3c–2e σ bonds. Fig. 4g shows eight 4c–2e σ bonds, four of which describe delocalized σ bonding between Rh and its neighboring B atoms and the other four describe delocalized σ bonding between the first and second B layers around the Rh atom. The Rh atom is coordinated with eight boron atoms *via* the four 4c–2e delocalized σ bonds and the two localized bonds in Fig. 4c and d. In addition to the four 4c–2e σ bonds, the bonding between the inner and outer B shells is described by the three 3c–2e σ bonds in Fig. 4f. The third row in Fig. 4 consists of five delocalized π bonds on the boron motif, rendering aromaticity to the quasi-planar isomer of RhB_18_^–^. We also find that there is even more charge transfer from Rh to the planar boron framework than to the drum framework, calculated to be 1.06 e at the PBE0 level.

While the delocalized π bonding in the quasi-planar RhB_18_^–^ is similar to that in the planar CoB_18_^–^ cluster,^24^ the coordination environment for Rh is different from that for Co. In the planar CoB_18_^–^ cluster, Co is coordinated by seven B atoms, whereas Rh is coordinated by eight B atoms in the quasi-planar RhB_18_^–^. Because of the smaller size of Co, the inner B_7_ ring has the right size to fit Co to give rise to a perfectly planar structure for CoB_18_^–^. However, the larger Rh requires a B_8_ ring for its first coordination shell in the quasi-planar RhB_18_^–^.

### 7.3. Competition between the drum and quasi-planar structures in metal-doped boron clusters

Small boron clusters have been found to be planar and electron delocalization in both the σ and π frameworks have been shown to be the major driving force.^3,4,6^ The tubular or drum structure was first shown to be viable for the neutral B_20_ cluster and was suggested to be the embryo for boron nanotubes.^8^ Ion mobility and DFT calculations suggested that cationic boron clusters (B_*n*_^+^) were all tubular for *n* > 15.^7^ However, for anionic B_*n*_^–^ clusters no tubular clusters have been observed up to *n* = 40.^5,6^ The curvature in tubular boron clusters may make the 2D electron delocalization unfavorable, so planar structures are favored. Clearly, the metal–boron interactions in CoB_16_^–^ and MnB_16_^–^ are critical in stabilizing the tubular structures. In CoB_18_^–^, because of the small size of Co atom the B_18_ drum motif is too large to allow effective Co–B interactions, resulting in the planar global minimum instead. Thus, there is a fine balance between M–B interactions in the drum isomers and 2D electron delocalization. In RhB_18_^–^, the slightly larger size of Rh makes the drum isomer competitive with the quasi-planar isomer and both are observed experimentally. Thus, it is conceivable that larger drums are possible with 5d, 6d or even lanthanide and actinide elements.

## 8. Conclusions

In summary, we have observed that in the RhB_18_^–^ cluster a perfect *D*_9d_ drum and a quasi-planar (*C*_s_) isomer are competing for the global minimum and both are observed experimentally. In the drum structure, the Rh atom features a record high coordination number of eighteen (CN = 18). The *C*_s_ quasi-planar isomer consists of a Rh atom coordinated with eight boron atoms in its first shell and an incomplete second shell of ten boron atoms. The quasi-planar isomer of RhB_18_^–^ is aromatic with 10 π electrons. The interactions between the Rh 4d orbitals and the B_18_ drum motif are favorable to make the *D*_9d_ drum isomer competitive with the quasi-planar isomer. The current work pushes the limit in coordination number in chemistry, suggesting that the size and bonding strength of the metal atom determines if a planar or tubular structure is more stable in the mid-sized metal-doped boron clusters. These insights can help design metallo-boronanotubes and metallo-borophenes by tuning the interaction between the metal and the boron atoms.
