# Supplementary material for: Competition between drum and quasi-planar structures in RhB18 –: motifs for metallo-boronanotubes and metallo-borophenes
Source: Chem Sci. 2016 Jul 25;7(12):7020–7. doi: 10.1039/c6sc02623k (PMC5355831; doi:10.1039/c6sc02623k)
Supplement: Supplementary file 1 [file SC-007-C6SC02623K-s001.pdf]

Electronic Supplementary Material (ESI) for Chemical Science.
This journal is © The Royal Society of Chemistry 2016

SUPPORTING INFORMATION

# Competition between Drum and Quasi-planar Structures in $RhB_{18}^-$: Motifs for Metallo-Boronanotubes and Metallo-Borophenes

Tian Jian[1†], Wan-Lu Li[2†], Xin Chen[2], Teng-Teng Chen[1], Gary V. Lopez[1],
Jun Li[2]*, and Lai-Sheng Wang[1]*

[1]*Department of Chemistry, Brown University, Providence, Rhode Island 02912 (USA)*

[2]*Department of Chemistry and Key Laboratory of Organic Optoelectronics & Molecular Engineering of Ministry of Education, Tsinghua University, Beijing 100084 (China)*

*E-mail: junli@tsinghua.edu.cn; Lai-Sheng_Wang@brown.edu

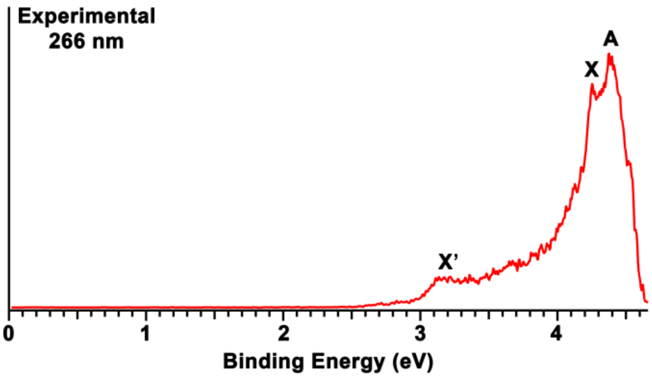


**Figure S1.** The photoelectron spectrum of $RhB_{18}^-$ at 266 nm (4.661 eV).

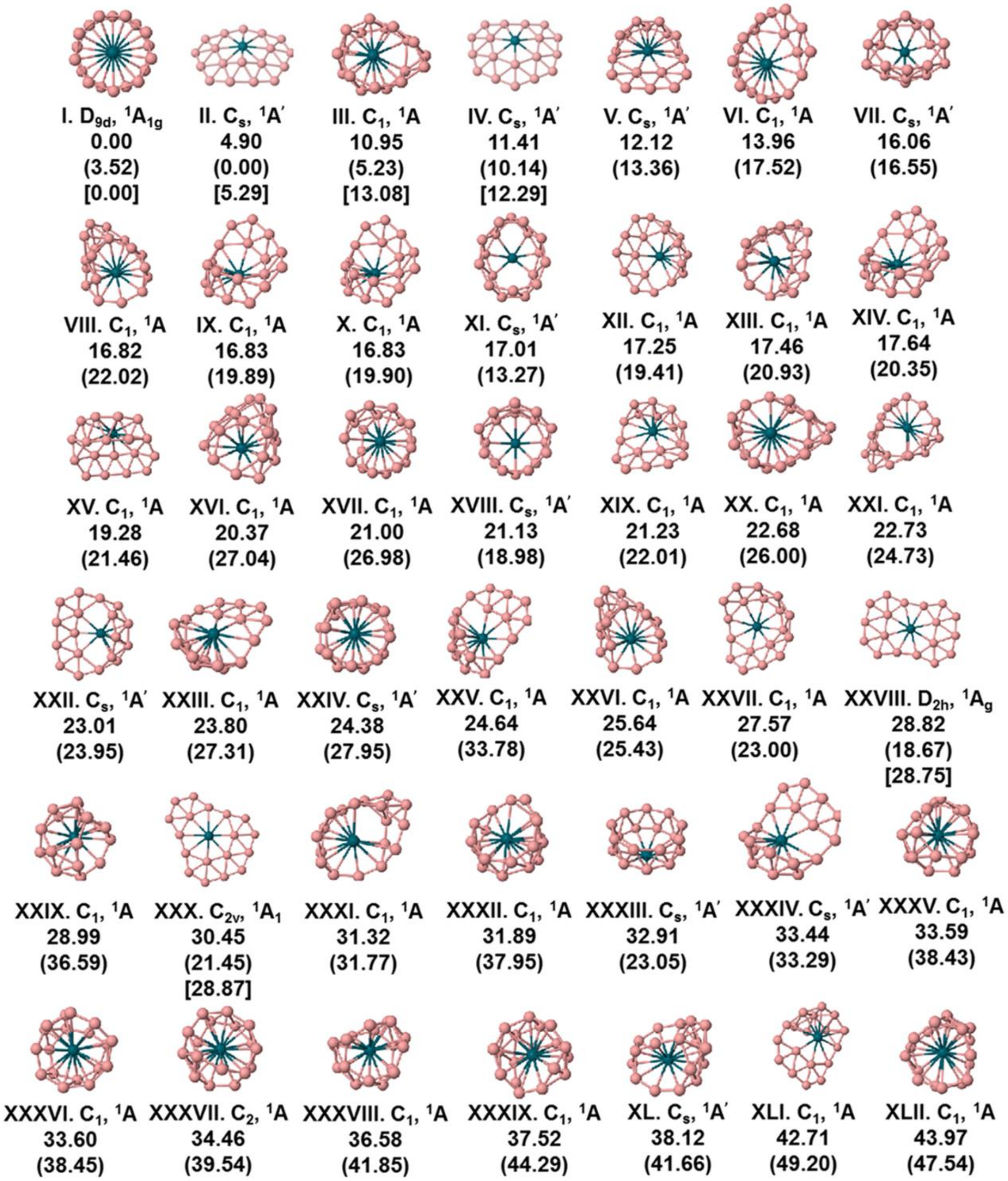


**Figure S2.** Relative energies in kcal/mol of the low-lying isomers of $RhB_{18}^{-}$ within 45.0 kcal/mol at PBE0/TZP, PBE/TZP in brackets and CCSD(T) levels in square brackets.

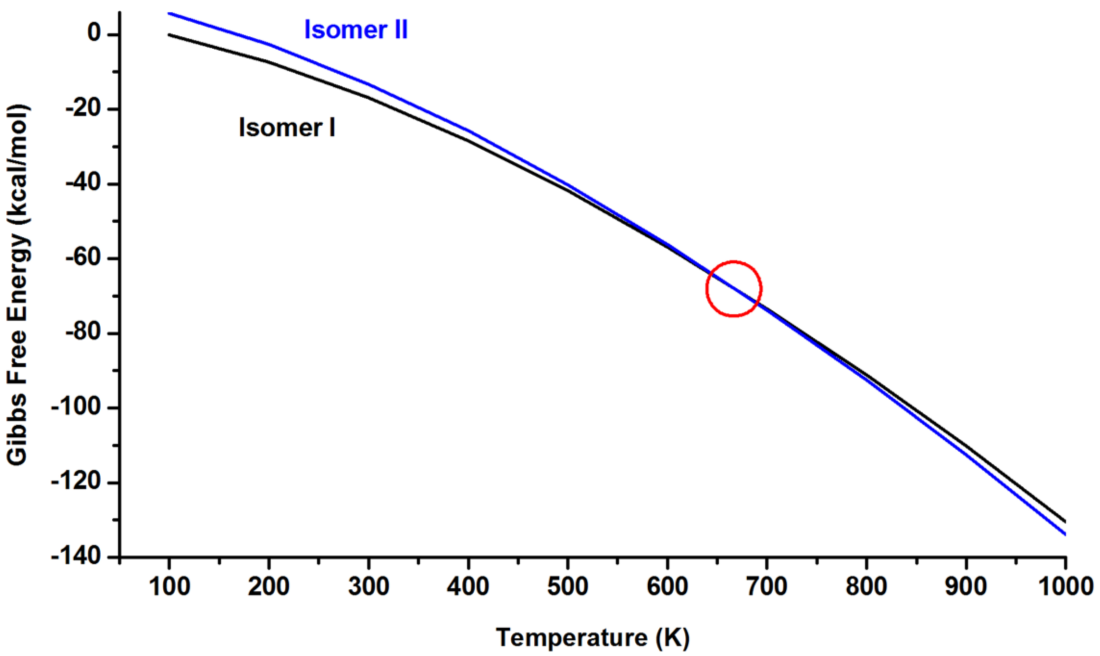


**Figure S3.** Relative Gibbs free energies (kcal/mol) of the *drum* isomer I and *quasi-planar* isomer II of $RhB_{18}^{-}$ in the temperature range from 100 to 1000 K at the PBE0/B/cc-pVTZ/Rh/ECP10MDF_VTZ level of theory. Note that the two isomers are nearly degenerate within a wide range of temperature; while PBE0 and CCSD(T) calculations predict that the isomer I is slightly more stable than isomer I, PBE ones predict the opposite.

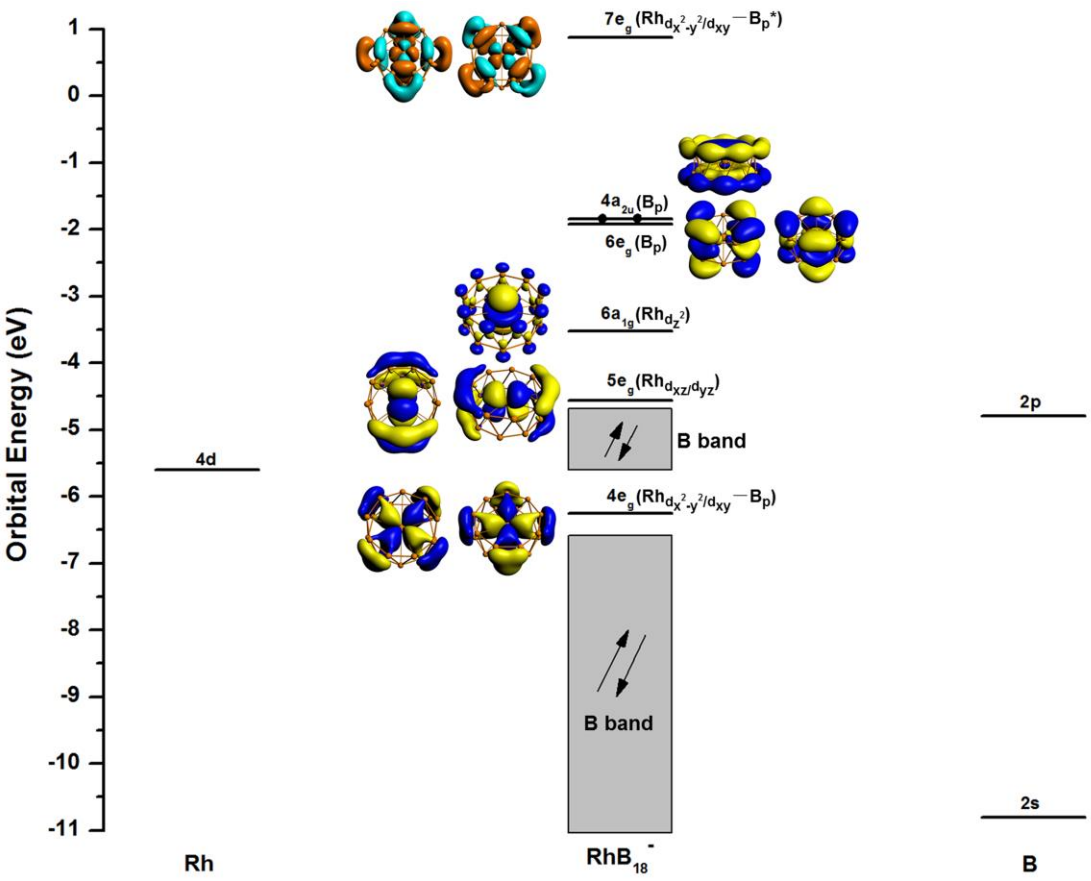


**Figure S4.** Kohn-Sham molecular orbital energy levels for the drum isomer of $RhB_{18}^{-}$ with selected molecular orbital contours.

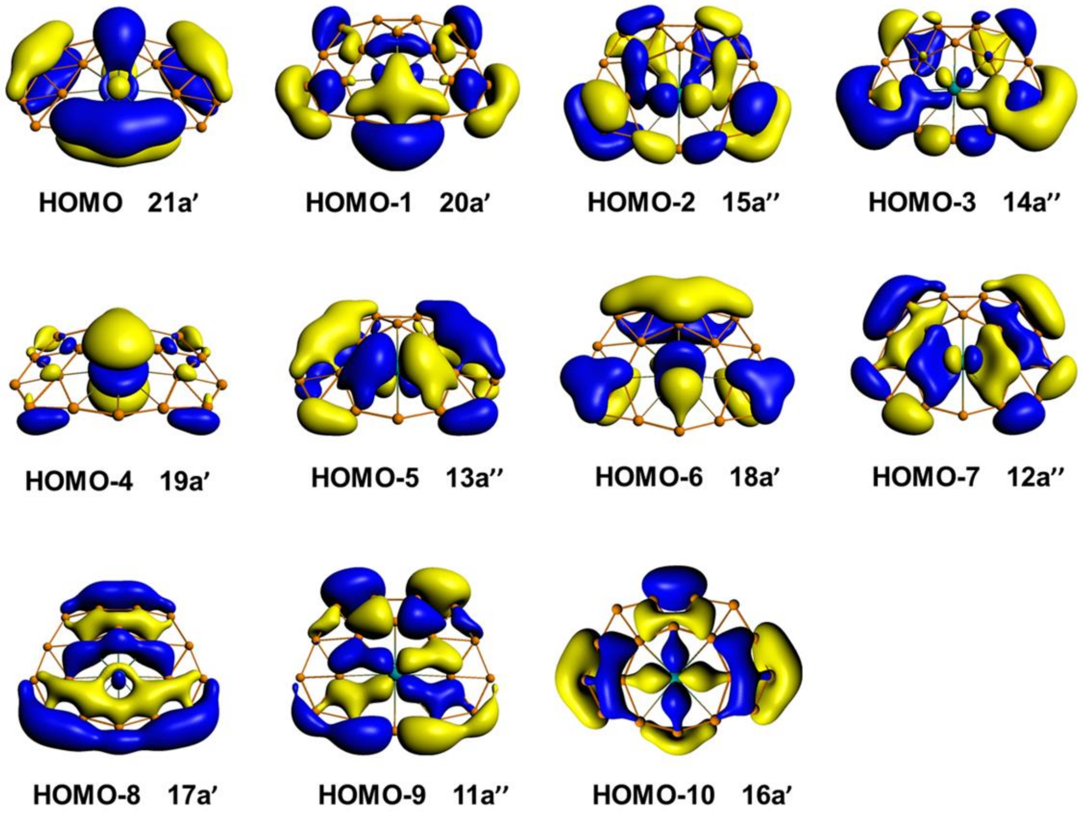


**Figure S5.** Valence Kohn-Sham molecular orbital contours for the quasi-planar isomer of $RhB_{18}^{-}$.

**Table S1.** Cartesian coordinates of the $D_{9d}$ drum and $C_s$ quasi-planar isomers of $RhB_{18}^-$ at the PBE0/TZP level of theory.

**$D_{9d}$ drum isomer:**

| | | | |
|---|---|---|---|
| 1.Rh | 0.000000 | 0.000000 | 0.000000 |
| 2.B | 0.000000 | 2.322405 | 0.827886 |
| 3.B | 1.492892 | 1.779109 | 0.827806 |
| 4.B | -1.492892 | -1.779109 | -0.827806 |
| 5.B | 0.000000 | -2.322405 | -0.827886 |
| 6.B | 2.287200 | 0.403328 | 0.827806 |
| 7.B | 1.492892 | -1.779109 | -0.827806 |
| 8.B | -2.287200 | -0.403328 | -0.827806 |
| 9.B | 2.287200 | -0.403328 | -0.827806 |
| 10.B | -1.492892 | 1.779109 | 0.827806 |
| 11.B | 2.011262 | -1.161203 | 0.827886 |
| 12.B | -0.794308 | -2.182437 | 0.827806 |
| 13.B | 0.794308 | -2.182437 | 0.827806 |
| 14.B | -2.011262 | 1.161203 | -0.827886 |
| 15.B | 0.794308 | 2.182437 | -0.827806 |
| 16.B | -0.794308 | 2.182437 | -0.827806 |
| 17.B | 2.011262 | 1.161203 | -0.827886 |
| 18.B | -2.287200 | 0.403328 | 0.827806 |
| 19.B | -2.011262 | -1.161203 | 0.827886 |

**$C_s$ quasi-planar isomer:**

| | | | |
|---|---|---|---|
| 1.Rh | -0.272015 | 0.450139 | 0.000000 |
| 2.B | 0.012811 | 0.464003 | -2.164864 |
| 3.B | 0.117565 | 2.172161 | -1.523826 |
| 4.B | 0.054250 | 2.600605 | 0.000000 |
| 5.B | 0.012811 | 0.464003 | 2.164864 |
| 6.B | -0.045689 | -1.100916 | 1.461179 |
| 7.B | 0.035083 | -2.740446 | 0.861958 |
| 8.B | -0.726098 | -1.508452 | 0.000000 |
| 9.B | -0.045689 | -1.100916 | -1.461179 |
| 10.B | 0.480422 | 0.410499 | -3.767007 |
| 11.B | 0.382113 | 1.784396 | 3.076109 |
| 12.B | 0.337400 | -1.090381 | 3.189937 |
| 13.B | 0.295152 | -2.469353 | -2.404338 |
| 14.B | 0.337400 | -1.090381 | -3.189937 |
| 15.B | 0.480422 | 0.410499 | 3.767007 |
| 16.B | 0.382113 | 1.784396 | -3.076109 |
| 17.B | 0.117565 | 2.172161 | 1.523826 |
| 18.B | 0.295152 | -2.469353 | 2.404338 |
| 19.B | 0.035083 | -2.740446 | -0.861958 |
